# Supplementary material for: Element-specific X-Ray detection of electron paramagnetic resonance in thin films of quantum bits
Source: Nat Commun. 2024 Nov 28;15:10313. doi: 10.1038/s41467-024-54586-3 (PMC11605076; doi:10.1038/s41467-024-54586-3)
Supplement: Supplementary file 1 — Supplementary Information [file 41467_2024_54586_MOESM1_ESM.pdf]

Element-Specific X-Ray Detection of Electron Paramagnetic Resonance in Thin Films of Quantum Bits

Andrin Doll,[1,2,a)] Zhewen Xu,[1] Vladyslav Romankov,[1] Giovanni Boero,[3] Stefano Rusponi,[4] Harald Brune,[4] Zaher Salman,[2] and Jan Dreiser[1,b)]
[1)]*PSI Center for Photon Sciences CPS, 5232 Villigen PSI, Switzerland*
[2)]*PSI Center for Neutron and Muon Sciences CNM, 5232 Villigen PSI, Switzerland*
[3)]*Microsystems Laboratory, École Polytechnique Fédérale de Lausanne, 1015 Lausanne, Switzerland*
[4)]*Institute of Physics, École Polytechnique Fédérale de Lausanne, 1015 Lausanne, Switzerland*

CONTENTS

Supplementary Note 1. Microwave setup 1
Supplementary Note 2. Fabricated samples 2
Supplementary Note 3. Sample temperature 2
Supplementary Note 4. High-flux XDEPR 3
Supplementary Note 5. Amplitude modulation 4
Supplementary Note 6. Chopping 5
Supplementary Note 7. Pulsed EPR at 6 GHz 6
Supplementary Note 8. Microwave field strength 8
Supplementary Note 9. Theory on g-free peak 10
References for Supplementary Information 13

SUPPLEMENTARY NOTE 1. MICROWAVE SETUP

This section describes the microwave resonator as well as the setup for spin excitation in further detail. A conceptual scheme of the microwave resonator was shown in the main text in Fig. 2a and its working principle was described in the methods section of the main text. This section describes the actual realization of the resonator on a base plate (21 mm width, 28 mm length, aluminium) that is compatible with standard sample holders used at the X-Treme endstation. Photographs of the deployed structure are shown in Supplementary Fig. 1a. The fully assembled probe is illustrated on the left and contains two half-wave microwave resonators (orange arrows) formed by the suspended aluminium bars with a central X-ray transmission hole (green arrows). The coupler used to feed one resonator with microwaves is shown in the small inset on the left. For variable coupling, the exposed plate of the coupler is brought to overlap with the open end of the resonator (magenta arrow), which forms an adjustable parallel plate capacitor. The coupling inside the X-Treme cryostat is monitored by microwave reflection measurements and through a camera. A photograph with the latter is shown on the right side in Supplementary Fig. 1a.

Supplementary Fig. 1a also shows a photograph of the bare sample plate with the microwave resonator unmounted (middle). The blue areas on the sample plate are the deposited phthalocyanine layers. The yellow kapton tape mounted between the two transmission holes holds two small flakes of 1,3-Bis(diphenylene)-2-phenylallyl (BDPA) : benzene (Sigma Aldrich, 152560) in proximity to each resonator (red arrows). These BDPA samples served as internal reference for electron spin resonance, which was monitored by the microwave signal reflected from the resonator.

The microwave setup is schematically depicted in Supplementary Fig. 1b and its components are listed in Supplementary Table 1. The setup incorporates the open-source LimeSDR software-defined radio spectrometer[1] that was op-

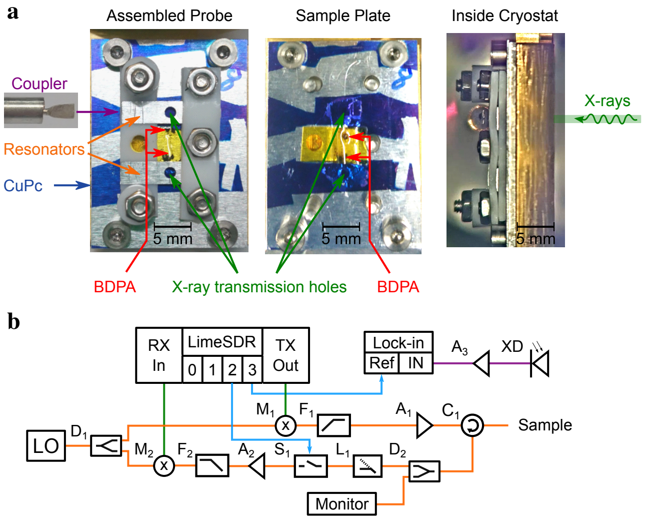

Supplementary Figure 1. Microwave resonator and microwave setup. **(a)** Realization of microwave resonator on 21x28 mm base-plate for operation in the X-Treme endstation. See text for description. **(b)** Schematic of the microwave setup, showing microwave connections (orange), lines at 2.5 GHz connecting to the LimeSDR (green), control and synchronization signals (blue), and the X-ray detection path (magenta). The components are described in Supplementary Table 1.

[a)]Electronic mail: andrin.doll@psi.ch.
[b)]Electronic mail: jan.dreiser@psi.ch.

Label	Device
A_1	Minicircuits, ZVE-3W-83+
A_2	Minicircuits, ZX60-83LN-S+
D_1	Minicircuits, ZX10-2-622-S+
D_2	Minicircuits, ZX10-2-183-S+, with 6 dB + 20 dB input attenuation
S_1	Minicircuits, ZASWA2-50DR-FA+
L_1	Minicircuits, VLM-83-2W-S+
C_1	JQL corp., JCC4000T8000S1R
M_1, M_2	Minicircuits, ZX05-14LH-S+
F_1, F_2	Minicircuits, VHF-4400+
LO	Era Instruments, EraSynth Micro
Monitor	Agilent Technologies, 8720ES
Lock-in	Anfatec, eLockIn203
A_3	Keithley, model 428
XD	Opto Diode, AXUV100G

Supplementary Table 1. Component listing of Supplementary Fig. 1 for XDEPR experiments.

erated at 2.5 GHz and frequency-translated to 7.5 GHz with a 5 GHz local oscillator (LO). Amplitude modulation at ω_{mod} and chopping at ω_{chop} have been performed digitally with the LimeSDR. For demodulation of the transmitted X-ray intensity detected by the diode XD, a reference signal at ω_{mod} was relayed from the LimeSDR to the lock-in amplifier.

SUPPLEMENTARY NOTE 2. FABRICATED SAMPLES

The set of samples prepared for XDEPR is summarized in Supplementary Table 2, listing four distinct CuPc samples, Cu-n with n = 1...4, and the VOPc film V-1. While the main text always explicitly indicates the used dilution and thickness, the sample names are used in the supporting information.

Name	Film composition	Dilution	Thickness [μm]
V-1	VOPc in $TiOPc$	10%	0.5
Cu-1	CuPc in H_2Pc	5%	1.0
Cu-2	CuPc in H_2Pc	10%	1.1
Cu-3	CuPc in H_2Pc	20%	0.4
Cu-4	CuPc in H_2Pc	10%	0.4

Supplementary Table 2. Thin film samples for XDEPR based on paramagnetic metal phthalocyanine molecules diluted into the respective diamagnetic host, specifying the dilution and film thickness.

SUPPLEMENTARY NOTE 3. SAMPLE TEMPERATURE

The sample temperature was inferred from magnetization curves $M(H)$ detected by X-ray transmission measurements. Representative magnetization curves for Cu-1 and V-1 are shown in Supplementary Fig. 2a and b, respectively. The blue data points show the experimental peak XMCD data, whereas the orange lines are fits to the data computed using iminuit, which is a python frontend to the Minuit library.[2] The trial function was $y = A(p-1)/(p+1) + A_0$ with $p = \exp\{(g\mu_B B)/(k_B T)\}$. In the latter expression, μ_B is the Bohr magneton and k_B the Boltzmann constant. For the electron g-factor, the same values as for the spectral simulations were used. The fitting parameters were therefore [A, A_0, T]. For CuPc, the fitted parameters were [0.92, -0.019, 6.6], while [1.13e-2, -8e-5, 4.9] resulted for VOPc. The resultant temperatures of $T = 6.6 \pm 0.5$ K and $T = 4.9 \pm 0.9$ K constitute the typical temperature range around 6 K in our experiments.

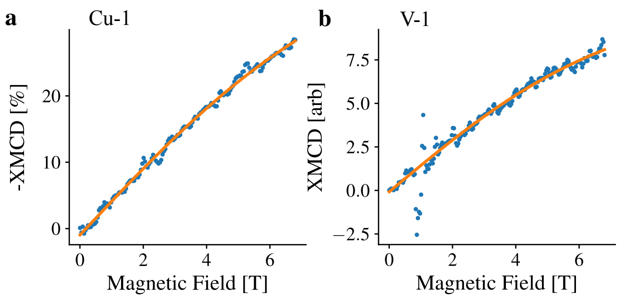

Supplementary Figure 2. XMCD magnetization curves up to 6.8 T. **(a)** Data in percentage scale with respect to L_3 edge for CuPc film Cu-1 (blue) and fit (orange) with temperature T = 6.6 ±0.5 K. The L_3 edge was probed at 931.1 eV, while the pre-edge was probed at 927.0 eV. **(b)** Unnormalized data for VOPc film V-1 (blue) and fit (orange) with temperature T = 4.9 ±0.9 K. Data points between 0.8 and 1.1 T were excluded from the fitting. Photon energies for maximum XMCD and the pre-edge were 516.72 eV and 515.0 eV, respectively. The L_3 edge amplitude was difficult to extract from these two photon energies, such that normalization was omitted.

XMCD spectra acquired at 3 T served as an estimation for the relative temperature increase due to (static) microwave sample heating. Example spectra zoomed onto the relevant L_3 edges are shown in Supplementary Fig. 3a and b for Cu-2 and V-1, respectively. The blue curves were acquired in absence of microwaves, whereas the oranges curves were acquired under microwave irradiation at 3 W and at 0.75 W, respectively. A reduction of the XMCD contrast upon microwave irradiation

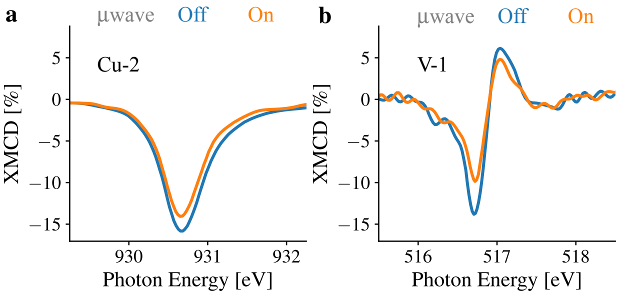

Supplementary Figure 3. Static microwave heating effect characterized by XMCD at 3 T and base temperature (6 K). **(a)** XMCD at L_3 edge of CuPc film Cu-2 in presence (orange) and absence (blue) of microwaves (7.36 GHz, 3 W, sinusoidal AM). **(b)** XMCD at L_3 edge of VOPc film V-1 in presence (orange) and absence (blue) of microwaves (7.54 GHz, 0.7 W, rectangular AM). All XMCD spectra are shown as precentage change with respect to the corresponding L_3 edge intensity.

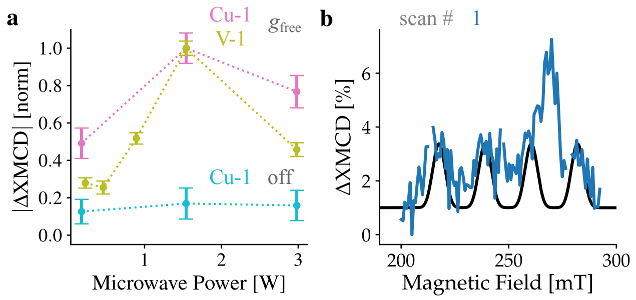

Supplementary Figure 4. Power-dependent and high-flux XDEPR. **(a)** Microwave power dependence of XDEPR signal at the g_{free} peak of Cu-1 (pink, 269 mT) and V-1 (olive, 271 mT) and at off resonance for Cu-1 (cyan, 340 mT). The contrast ΔXMCD is normalized to the maximum value for each sample. The error bars represent the noise level in demodulated time-domain data. **(b)** XDEPR spectrum of Cu-2 acquired during 40 minutes at an enhanced photon flux of 100 ph/ms/μm^2 (blue). The simulated XDEPR spectrum (black) has the same parameters than for Fig. 2b in the main text, plus an additional vertical offset by 1 % to account for the non-resonant thermal background. See also Supplementary Fig. 5a, showing the same experimental data and subsequent scans.

is evident from the datasets. Since the magnetization follows the temperature linearly at the field strength of 3 T, the microwave heating effect is below a factor of two in these two examples. Accordingly, the sample temperature did not rise beyond 10 K, which is considered an important prerequisite for the experimental observation of XDEPR.

Further data on the critical balance between microwave heating and XDEPR signal strength are shown in Supplementary Fig. 4a, which shows ΔXMCD as a function of microwave power at the g_{free} peak of CuPc (blue) and VoPC (green) as well as the non-resonant background for CuPc (orange). Best performance was achieved at a microwave power of 1.5 W, whereas the largest power of 3 W caused adverse sample heating.

While sample heating limits the applicable microwave power, and therefore the attainable signal-to-noise ratio of XDEPR, the X-ray photon flux is another parameter that determines the signal-to-noise ratio. The following section presents XDEPR data at a ten times larger photon flux.

SUPPLEMENTARY NOTE 4. HIGH-FLUX XDEPR

The influence of the photon flux onto XDEPR data was a very critical factor for the observation of XDEPR spectra. The main reason is that XAS/XMCD properties are not significantly altered by a *large* photon flux on the order of 100 ph/ms/μm^2, whereas the XDEPR spectra can only be observed within the first few tens of minutes.

In principle, the larger photon flux significantly increases the signal-to-noise ratio, as is shown in Supplementary Fig. 4b, where a single 40-min field scan (blue) shows a clear signature of the expected hyperfine structure of CuPc (black). However, the XDEPR spectrum was not preserved under these

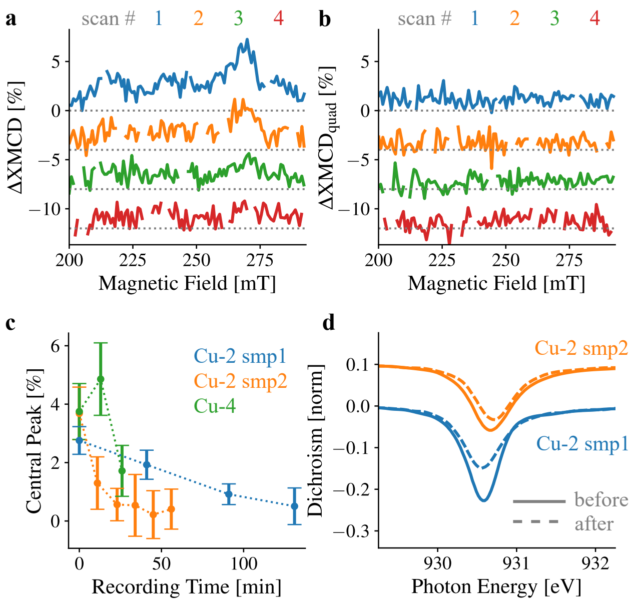

Supplementary Figure 5. Suppression of XDEPR spectra at large photon flux. **(a)** Series of XDEPR spectra of Cu-2 with vertical displacement according to the recording sequence, which initiated with the blue curve. **(b)** Quadrature AC component that has a 90° phase shift to microwave modulation for the data shown in panel (a). **(c)** Amplitude of the central XDEPR peak for successive scans on two different Cu-2 samples (blue and orange) and on a Cu-4 sample (green). Dotted lines are guides to the eye and the error bars indicate the noise level in the XDEPR spectra. The four blue data points were extracted from the four spectra in panel (a). The error bars represent the noise level in demodulated time-domain data. **(d)** XMCD at L_3 edge before (solid) and after (dashed) recording the series of XDEPR spectra in panel (c). The color code is identical to panel (c), with the orange curve vertically offset by 10%, and the magnetic field was 3 T.

conditions, as summarized in Supplementary Fig. 5. Panel (a) shows the experimental ΔXMCD data of four subsequent field scans, each having a duration of 40 minutes. Each scan is vertically offset, color-coded, and complemented by the corresponding zero level (dotted). The suppression of the XDEPR signal is clearly visible.

Besides the XDEPR signal that is demodulated at the same phase as the microwave envelope modulated at ω_{mod}, the quadrature component that is demodulated with a phase shift of 90° with respect to the microwave envelope is shown in Supplementary Fig. 5b. While the XDEPR contribution is not visible in this contribution, all scans have a non-zero baseline. We attribute this baseline to the dynamic variation of the temperature with ω_{mod}. Further examples are found in the main text, as for instance by the non-zero baseline in XDEPR spectra or even more clearly by largely off-resonant XDEPR at 3 T (magenta in Fig. 2e). The phase shift of the heating pathway with respect to the EPR pathway is in agreement with a different mechanism underlying the variation of the XMCD-detected spin polarization. While the EPR pathway immediately follows the modulation envelope (see also Supplementary Note 5 below), the heating pathway lags behind due to

the transient response of the cryostat to microwave heating at ω_{mod}.

With the baseline level in the quadrature component related to microwave heating, the loss of the XDEPR spectrum for the in-phase component can be attributed to a variation of EPR properties. Essentially, a non-zero baseline level due to microwave heating implies that (i) the microwave driving amplitude and (ii) the magnetic moment of the sample were conserved throughout the experimental series. A trivial explanation of the observed loss of the XDEPR spectrum is therefore excluded. Examples include X-ray damage causing a reduced moment at the metal centre or drift in microwave drive conditions or sample temperature.

Besides the experimental series on Cu-2 discussed above, two other series were acquired and compared in Supplementary Fig. 5c via the intensity of the principal XDEPR peak as a function of acquisition time. The blue data points represent the four scans of Cu-2 illustrated in panels (a) and (b). The orange data points are from another Cu-2 sample acquired with faster field scanning, namely from the second microwave resonator available on the same sample holder. The green data points originate from Cu-4, which served as a third independent reference that was interrupted after three scans due to the onset of XDEPR suppression in primary data. As is readily seen, all three series show a decay of the XDEPR intensity. The timescale of the decay appears to differ among different series and might depend on the total X-ray dose absorbed during a particular series. These differences were not investigated further, since XDEPR data could be acquired over several hours by a reduction of the photon flux from 100 ph/ms/μm^2 down to 10 ph/ms/μm^2.

To further underline that the magnetic moment of the Cu spins was conserved despite the suppressed XDEPR spectrum, Supplementary Fig. 5d shows XMCD spectra of the L_3 edge at 3 T before (solid) and after (dashed) the corresponding experimental session with the two Cu-2 samples (blue and orange, as in panel c). While the blue curves point at a reduction of XMCD that could be related to a change in either sample environment or properties throughout the entire experimental session that lasted 20 h, the orange curves from a shorter experimental session (7 h) exhibit only a small change in XMCD, which rules out damage to the sample.

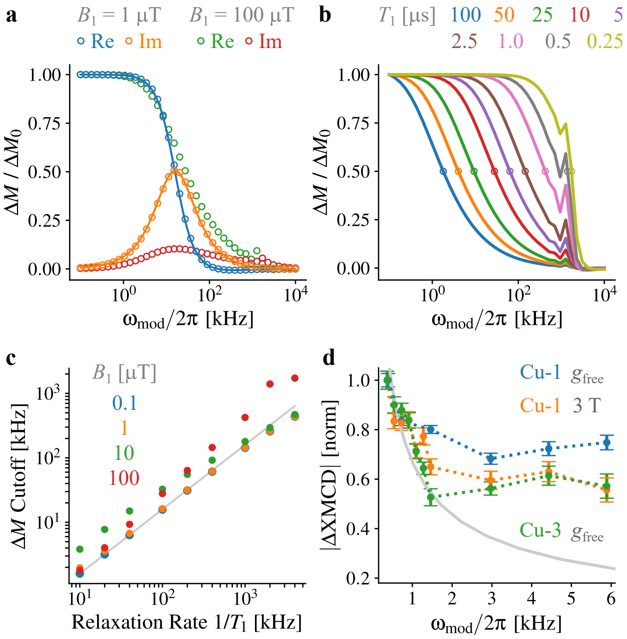

Supplementary Figure 6. Simulated dependence of ΔM on amplitude modulation frequency ω_{mod} for different drive and T_1 relaxation parameters, with fixed T_2 = 200 ns. **(a)** Simulated complex valued data points (dots) using T_1 = 10 μs for weak drive with B_1 = 1 μT ($\mathfrak{Re}$ blue, $\mathfrak{Im}$ orange) and for a stronger drive with B_1 = 100 μT ($\mathfrak{Re}$: green, $\mathfrak{Im}$: red). The blue and orange solid lines that virtually overlap with the simulated data points are the T_2-dependent analytical prescription in the weak-drive limit.[4] **(b)** Influence of T_1 on in-phase ΔM response ($\mathfrak{Re}$ in panel a) with B_1 = 100 μT. The dots indicate the ΔM cutoff frequencies where the AC magnetization is reduced to 50 % due to the inability of the magnetization to follow the drive envelope. **(c)** Simulated dependence of ΔM cutoff frequency on relaxation rate $1/T_1$ for the color-coded B_1 driving strengths. The gray line is the linear slope with cutoff corresponding to $1/(2\pi T_1)$ that is expected in the weak-drive limit.[4] **(d)** Experimental $|\Delta\mathrm{XMCD}|$ as a function of the modulation frequency ω_{mod} at the g_{free} resonance of Cu-1 (blue) and of Cu-3 (green) as well as 3 T for Cu-1 (orange). The data are normalized to the first data point in each series. The gray line shows a simulation for T_1 = 0.5 ms , T_2 = 200 ns and B_1 = 100 μT scaled up by a factor of 3 in order to overlay with the initial part of experimental data. The error bars represent the noise level in demodulated time-domain data.

SUPPLEMENTARY NOTE 5. AMPLITUDE MODULATION

This section provides further details on the simulations and experimental data with the employed amplitude modulation schemes. The principal modulation scheme in this work is the microwave envelope modulation at ω_{mod} which results in a modulation of the spin polarization with amplitude ΔM. This modulation scheme has been analyzed previously in the context of longitudinal detection of magnetic resonance (LOD),[3] including analytical solutions involving both T_1 and T_2 relaxation times.[4] However, the analysis was tied to the limit of weak driving amplitude, which does not correspond to our experimental conditions. Moreover, since these experiments rely on a coil to detect the AC modulation of ΔM, the analytical solutions correspond to the time-domain derivative of ΔM.[3] Consequently, analytical solutions for ΔM are obtained by time-domain integration of the reported equations.[4]

The analytical dependence of ΔM on ω_{mod} is plotted by the blue and orange solid lines in Supplementary Fig. 6a, which depict the real and imaginary components, respectively. These solid lines are superimposed by round circles of the same color that were obtained by numerical computation of the steady state solutions to the Bloch equations (see Methods section). The numerical and analytical results assumed relaxation times T_1 = 10 μs and T_2 = 200 ns. Since the analytical results are valid within the weak-drive limit only ($B_1 \ll (g\mu_{\mathrm{B}}T_2)^{-1} \approx 200$ μT), a correspondingly small drive amplitude of 1 μT has been assumed in the simulation. The

excellent agreement between the analytical prescription and the numerical simulation serves as validation for the numerical simulation approach. The departure from the weak-drive limit is shown by another simulation at a larger amplitude of 100 μT, which is shown by the green and red dots in Supplementary Fig. 6a for the real and imaginary parts, respectively.

The in-phase component of ΔM shows the cutoff behavior when the modulation period becomes fast compared to T_1, such that the magnetization can no longer follow the microwave envelope and becomes saturated. For weak driving, the quadrature component of ΔM coincides with the real part at the cutoff frequency where $\omega_{\mathrm{mod}} = T_1^{-1}$.[4] For the simulation with larger drive amplitude, a cutoff in the in-phase component is still evident, whereas the phase shift becomes smaller.

The influence of T_1 onto the cutoff behavior of the in-phase component of ΔM is shown in Supplementary Fig. 6b, where the different colors indicate T_1 times ranging from 100 μs to 250 ns. These simulations were performed for a large drive, e.g with T_2 = 200 ns and B_1 = 100 μT. All curves exhibit a kink-feature in the lower MHz regime, which we attribute to coincidence of the Rabi oscillation frequency at 3 MHz and ω_{mod}. The round circles indicate the cutoff frequencies where ΔM was reduced by 50 %.

These cutoff frequencies are furthermore plotted as a function of the relaxation rate T_1^{-1} in Supplementary Fig. 6c, where the red data points correspond to the simulation with the 100 μT drive from panel (b). The other colors originate from simulations with weaker drive amplitudes, as indicated by the legend. The solid gray line is the analytical weak-drive limit that is reproduced by the simulations with small drive amplitudes. The shift to slightly larger cutoff frequencies when increasing the drive amplitude, as evident already in Supplementary Fig. 6a, is here reproduced for a broad range of parameters.

In summary, we conclude that the $\Delta M(\omega_{\mathrm{mod}})$ relation permits viable insight into longitudinal relaxation times, in line with previous LOD studies analyzed in the limit of a weak drive.[3–5] In principle, direct access to the change in the spin polarization even renders the $\Delta M(\omega_{\mathrm{mod}})$ relation suitable for a broader range of relaxation times than for LOD, since the derivative of ΔM is difficult to capture by a pickup coil at low frequencies. For this reason, LOD has initially been developed for the study of species with very fast T_1 times.[3,4] However, as encountered in our study, the range of experimentally accessible T_1 values might be limited by instrumental restraints on the detector. In fact, the employed large-area diode showed cutoff behavior starting from 9 kHz, which was the reason for limiting the largest experimental modulation frequency to 6 kHz, where the detector response was still flat.

Another instrumental feature in our experiments is related to the low-frequency response of the detector. Exemplary experimental data are shown in Supplementary Fig. 6d, illustrating $\Delta M(\omega_{\mathrm{mod}})$ for the principal g_{free} peak of Cu-1 (blue) and of Cu-3 (green) as well as largely off resonance to Cu-1 at 3 T (orange), which arises from dynamic microwave heating. In principle, the curves from Cu-1 have already been shown in the main text in Fig. 2e. Here in Supplementary Fig. 6d, the same curves are plotted upon normalization by the lowest-frequency data point. As is readily seen, all curves exhibit an initial drop in the signal as the frequency increases. Instead of being related to the relaxation dynamics of ΔM, we attribute this low-frequency drop to the response of the detector. For reference, the gray curve is a simulation of $\Delta M(\omega_{\mathrm{mod}})$ that follows this initial part of the decay for T_1 = 0.5 ms, T_2 = 200 ns and B_1 = 100 μT. In analogy to the discussion in the main text, where the gray curve in Fig. 2e assumed T_1 = 100 μs, the experimental data do not follow this decay to higher modulation frequencies, where a flat response reminiscent of short T_1 relaxation times is observed.

SUPPLEMENTARY NOTE 6. CHOPPING

As explained in the main text, an additional modulation at ω_{chop} was introduced in order to access T_1 dynamics beyond the bandwidth of the employed detector. Both in the experiments and in the simulations, the detected quantity remains $\Delta M(\omega_{\mathrm{mod}})$ as in the modulation pathway discussed above in Supplementary Note 5. Information on T_1 is, however, accessed by variation of ω_{chop}, while maintaining ω_{mod} at a constant frequency below ω_{chop}. In the following, characteristic

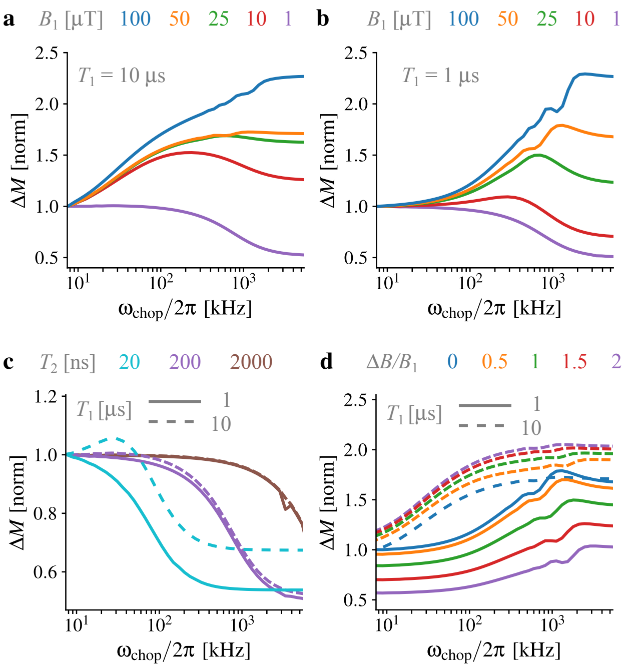

Supplementary Figure 7. Simulated dependence of ΔM on chopping frequency ω_{chop} for different drive and relaxation parameters. **(a,b)** Progression in microwave driving strength B_1 over two orders of magnitude (see legend) for T_1 = 10 μs in panel (a) and for T_1 = 1 μs in panel (b), using T_2 = 200 ns. **(c)** Influence of T_2 at small drive (B_1 = 1 μT) for relaxation time T_2 that is an order of magnitude slower (brown) and faster (cyan) than in all other simulations (purple). Solid and dashed lines represent different T_1 times. **(d)** Progression in detuning ΔB from the resonance (see legend, B_1 = 50 μT, T_2 = 200 ns), where each of the two sets with different T_1 is normalized by ΔM with on-resonance drive at the smallest illustrated ω_{chop}.

features in the resultant $\Delta M(\omega_{chop})$ relation are outlined by numerical simulations.

A first important aspect is the influence the driving amplitude B_1, which is illustrated in Supplementary Figs. 7a and b for T_1 = 10 µs and T_1 = 1 µs, respectively. The curves are colored according to the simulated driving amplitudes in the legend. In general, all the curves with $B_1 >= 10$ µT show a frequency-progressive initial rise of $\Delta M(\omega_{chop})$ which furthermore increases with B_1. As explained in the main text, this increase is related to the saturation on the timescale of the chopping period. Furthermore, all the simulated curves have a flattened or even decaying high-frequency tail.

We noted that the onset of the high-frequency tail depended on T_2, which is illustrated in Supplementary Fig. 7c at the lowest considered driving amplitude of 1 µT for both T_1 = 1 µs (solid) and T_1 = 10 µs (dashed) for different color-coded T_2 values. The purple curves are the same as those shown in panels (a) and (b). The virtual overlap of the two purple curves confirms that the influence of T_1 is marginal at these very small driving amplitudes. However, the cutoff of $\Delta M(\omega_{chop})$ strongly depends on T_2. While these simulations suggest that information on T_2 could be obtained from $\Delta M(\omega_{chop})$ with a weak drive, it should be noted that this represents a very small amplitude well below the driving strength estimated in our experiments (see also Supplementary Note 8).

Since the simulations were so far all for a drive that is in exact resonance with a two-level system, the off-resonance behavior of $\Delta M(\omega_{chop})$ was considered. Of particular relevance was whether the frequency-progressive increase of $\Delta M(\omega_{chop})$ is altered by off-resonant excitation. The simulation results are shown in Supplementary Fig. 7d for both T_1 = 1 µs (solid) and T_1 = 10 µs (dashed) for different color-coded resonance offsets ΔB. As seen in the simulated curves, off-resonant excitation is mainly reducing the overall amplitude of ΔM due to the less efficient drive into saturation. On the contrary, the increase of ΔM with ω_{chop} originates from the extent of saturation recovery in absence of the drive. One would therefore expect that an increase of ΔM with ω_{chop} can also be observed for the inhomogeneously broadened lines encountered experimentally.

Given the critical influence of the drive and relaxation parameters on the shape of $\Delta M(\omega_{chop})$, precise estimations of the underlying parameters seem rather difficult with the chopping experiments, especially in the case at hand where T_1, T_2, and $1/\omega_1$ become comparable. As a counter-example, the influence of T_1 on the cutoff frequency with envelope modulation exclusively at ω_{mod} was found to be more robust against variations in drive (see Supplementary Fig. 6c) and T_2 (data not shown). Nevertheless, in a situation where instrumental limitations restrain the range of feasible modulation frequencies ω_{mod}, additional insight can be gained by the introduction of ω_{chop}. In essence, the experimental ΔXMCD(ω_{chop}) in Figs. 3cd in the main text complement the absence of a clear cutoff in the ΔXMCD(ω_{mod}) data and restrain T_1 to the lower microsecond range, which is much shorter than the T_1 times from pulse EPR on CuPc.

SUPPLEMENTARY NOTE 7. PULSED EPR AT 6 GHZ

The pulsed EPR spectrum shown in Fig. 1b in the main text was acquired on a dedicated experimental setup that is described in the following. The microwave spectrometer was the same as the one illustrated in Supplementary Fig. 1b, where instead of the 3 W continuous-wave (CW) amplifier A_1, a 100 W CW amplifier with frequency range from 2 - 6 GHz was used (Minicircuits, HPA-100W-63+). For operation of this amplifier in a pulsed EPR setup, its input was gated by an additional switch of the same type as S_1. The high-power output of the amplifier was protected by a terminated circulator (Uiy Inc, BCC3030A2T6NF). After this circulator, the noise of the amplifier output injected during echo detection into the receiver was gated by a fast switch rated for such high powers (Qorvo, TGS2355-SM).

With the highest frequency tied to 6 GHz by the frequency range of the high power amplifier, the pulsed EPR experiments used a different half-wave microwave resonator to couple to the spins. The resonator is based on a commercially available coplanar waveguide board (Southwest Microwave, B4350-30C-50) and is shown in Supplementary Fig. 8a. The photograph on the left shows the waveguide board with its feeding port (blue arrow). A half-wave resonator was created by opening a gap in the waveguide and the position of maximum microwave magnetic field at its center is indicated by the dashed magenta line. In order to couple the feedline to this resonator, a coupler stick featuring a small electrode was used (see red arrow). The electrode was brought in direct contact with the feedline, so as to form a mechanically adjustable capacitor between the feedline and the resonator.

The fully assembled microwave probe is shown on the right in Supplementary Fig. 8a. As seen at the very top, the coplanar feedline was interfaced to a coaxial connector via an appropriate adapter (Southwest Microwave, 1093-01A-5). The resonator was fully covered by the sample plate with the Cu-1 film facing the board. Teflon spacers between the board and the sample plate established a separation on the order of 0.5 mm. At the position of the sample (magenta arrow), a small flake of BDPA has furthermore been placed between the sample plate and the resonator. This BDPA flake served as calibration for the magnetic field and for the pulse flip angles. Note that exactly the same sample plate as used for XDEPR was incorporated into the resonator. For the investigated Cu-1 sample, the pulse EPR spectra therefore originated from exactly the same sample as for XDEPR.

For operation at a temperature of 5 K, the resonator structure was positioned inside a helium flow cryostat (Oxford instruments, CF935) via a semi-rigid coaxial cable of appropriate length (Coax Japan Co., SC-358/50-SSS-SS). An electromagnet provided the static magnetic field pointing outside of the resonator board plane.

The microwave resonator exhibited a rather pronounced background signal at 5 K, as is explained in Supplementary Fig. 8b. The plot shows the amplitude of the spin echo as a function of the amplitude of the driving pulses at two field positions. The first field position (blue) was in resonance to the BDPA reference sample, where as the second field posi-

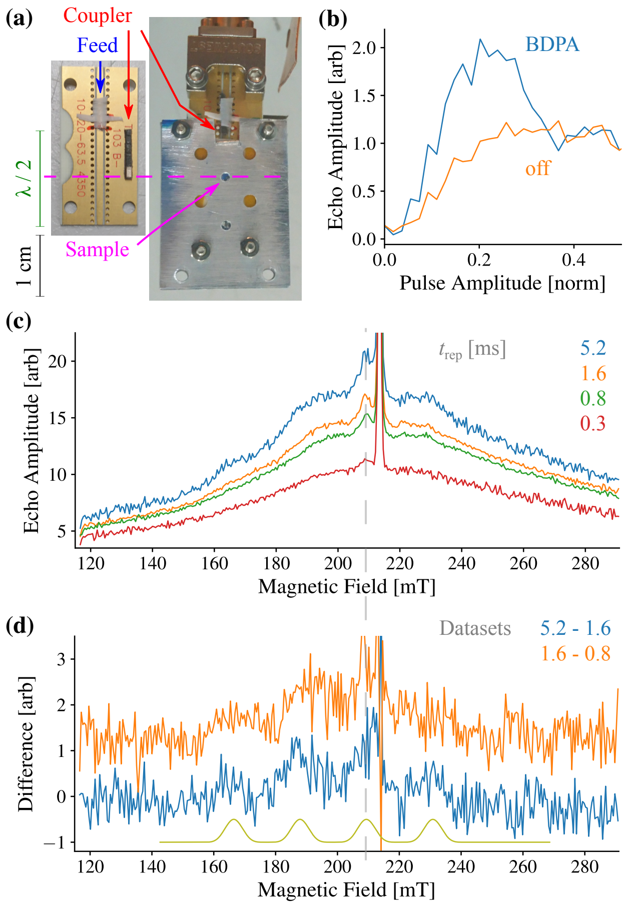

Supplementary Figure 8. Pulsed EPR of Cu-1 sample at 6 GHz and 5 K. **(a)** Photographs of half-wave microwave resonator and sample, where the coplanar waveguide board is shown on the left and the fully assembled probe on the right. The annotations mark the feedline (blue), the coupling mechanism (red), the longitudinal extension of the half-wave resonator (green) as well as its center position of maximum magnetic field (dashed magenta), and the position of the sample (magneta). See text for further descriptions. **(b)** Dependence of the echo amplitude on excitation pulse amplitude recorded in resonance to the BDPA reference (blue) as well as at a 5 mT lower field (orange). See text for pulse sequence parameters. **(c)** Field-swept spectra of Cu-1 recorded over a range of 175 mT with 351 points for different repetition times t_{rep}, as indicated by the color coding in the legend. See text for pulse sequence parameters. **(d)** Difference between EPR spectra of panel (c) scaled to the same integrated amplitude, where the underlying subtraction scheme is described in the legend with reference to the repetition times of corresponding spectra. The olive curve shows the simulated CuPc spectrum at the relevant microwave frequency of 6 GHz. Note that the pulsed EPR spectrum shown in Fig. 1b corresponds to the data shown in blue with an additional three-point binning of the data along the field axis to improve visual clarity.

tion (orange) was 5 mT lower and therefore off resonance to the BDPA reference. The Hahn echo pulse sequence consisted of two pulses with durations of 33 ns and 66 ns, respectively, with a delay of 396 ns between starting pulse flanks. A phase cycle with a total of 16 phase constellations according to the exorcycle was employed.[6] The repetition time of the experiment was 2.1 ms and each phase constellation was averaged 100 times.

The echo amplitude from BDPA shows a clear maximum for a pulse amplitude about 0.2 (relative to full-scale). For this pulse amplitude, the microwave pulses realize the spin rotations of $\pi/2$ and π that maximize the Hahn echo amplitude. When doubling the pulse amplitude to around 0.4 (relative to full-scale), one would expect that the echo from BDPA vanishes, since the first pulse becomes a π pulse that inverts the spin polarization, instead of exciting a coherent superposition state. As seen in the data, however, a non-zero echo amplitude is observed. The origin of this contribution becomes evident when comparing to the off-resonant data, where a gradual increase to a plateau is observed. We attribute this off-resonant contribution to background impurities that are distributed throughout the entire length of the half-wave resonator. The spatial distribution results in the absence of a distinct maximum in the off-resonant curve. In essence, spins distributed throughout the resonator experience different microwave field strengths. The optimum rotation angles of $\pi/2$ and π can thus not be fulfilled simultaneously for such distributed spins, which explains the experimentally observed plateau. This background signal is probably related to the substrate used in the coplanar waveguide board.

Extraction of the echo signal from the thin-film Cu-1 sample required a background suppression method, which was accomplished here by variation of the repetition time t_{rep} of the pulse sequence. In general, the dependence of the echo amplitude on t_{rep} is determined by T_1. In the limit where $t_{rep} \gg T_1$, thermal equilibrium is established prior to echo generation, which maximizes the echo signal. If t_{rep} is on the same order or even shorter than T_1, on the contrary, the (repeatedly averaged) spin echo probes a partially saturated state that results in a smaller echo amplitude. Background suppression based on variation of the repetition time therefore assumes that the background and the signal of interest have largely different T_1 times. Other than the variation in t_{rep}, the echo sequence consisted of two 33 ns long pulses with amplitudes optimized at the BDPA reference and with a delay between starting pulse flanks of 363 ns.

Experimental EPR spectra are shown in Supplementary Fig. 8c with color-coding according to the t_{rep} values in the legend. Due to the partial saturation explained above, there is a general trend to larger echo amplitudes when prolonging t_{rep}. At the shortest repetition time of 0.3 ms (red), t_{rep} is comparable to the T_1 time expected for Cu-1 based on the literature.[7] One would therefore expect that the contribution from CuPc is largely saturated for this setting. For the longest t_{rep} of 5.2 ms, on the contrary, CuPc is expected to contribute. When looking at the shape of the raw spectra, additional features can indeed be identified. At field positions around 165 mT and 230 mT, for instance, additional spectral peaks emerge when comparing the blue to the red spectrum.

Besides the changes in spectral features, the field-swept spectra also display quite different overall noise levels. While the green and orange curves have the best quality, the noise level is slightly stronger for the red and much stronger for the

blue curve. These difference are related to signal averaging. In order of ascending repetition times, the number of echo averages per field point were 120k, 168k, 144k, and 27k, respectively. The smaller number for the blue data set with the longest repetition time is explained by the overall acquisition times of 3.5 hrs, 16 hrs, 27 hrs, and 15 hrs, respectively.

For a more quantitative identification of the slowly relaxing contributions in the EPR spectra, the spectra were all scaled to the same integrated intensity and then the difference was formed between two scaled datasets. Spectra obtained in this way are shown in Supplementary Fig. 8d, where the blue curve shows the difference between the scaled spectra acquired with t_{rep} = 5.2 ms and t_{rep} = 1.6 ms and the orange curve correspondingly for data acquired with t_{rep} = 1.6 ms and t_{rep} = 0.8 ms. For visualization purposes, the orange curve has been scaled by a factor of two and displaced vertically. The plot furthermore shows the simulated CuPc spectrum at the microwave frequency of 6 GHz (olive). As is readily seen when comparing the blue curve to the spectral positions of the four-line spectrum of CuPc, the expected spectral features indeed emerge out of the noise floor. A similar, but slightly less clear assignment is possible with the orange curve.

Besides the CuPc spectral features, the difference spectra contain other spectral components. First, there is a contribution around g_{free} due to the BDPA reference sample that has been placed inside the resonator for calibration purposes. Its spectral position is readily inferred from the large peak in Supplementary Fig. 8c. This feature at g_{free} is, however, much narrower than the very broad and centered g_{free} peak observed in all XDEPR spectra. Second, there is probably a contribution from an overlapping peak on the third hyperfine line which is marked by the dashed gray line. The reason for this assumption is the peak feature in the otherwise broad and feature-less spectrum acquired with t_{rep} = 0.3 ms (red). Exactly the same spectral feature has also been observed when trying to acquire pulsed EPR spectra of V-1, which did not provide any spectral signature beyond the background.

Further potential to experimentally improve the background correction notwithstanding, the data approve that the native EPR properties of Cu-1 are in accordance with literature.[7] Of particular relevance is the clear emergence of the highest- and lowest-field hyperfine line when increasing t_{rep} from 1.6 ms to 5.2 ms. Such a behavior cannot be explained when assuming a T_1 relaxation time well below 100 µs, as deduced from XDEPR on the highest-field hyperfine line.

SUPPLEMENTARY NOTE 8. MICROWAVE FIELD STRENGTH

The microwave field strength of the XDEPR microwave resonator at 7.5 GHz was calibrated by means of CW EPR spectroscopy. The CW EPR experiments were performed at room temperature on a dedicated setup with an electromagnet. The microwave spectrometer corresponds to the one shown in Supplementary Fig. 1b. The microwave resonator and coupling mechanism were an exact structural analogue to the XDEPR resonator shown in Supplementary Fig. 1a. A saddle-shaped coil provided magnetic field modulation at 110 kHz that is required for CW EPR. Further methodological details on the acquisition of CW EPR spectra with the employed open-source spectrometer are given elsewhere.[1]

The sample consisted of a set of grains of the BDPA radical at the centre of the resonator. A low-power CW EPR (derivative) spectrum of this sample is shown in Supplementary Fig. 9a, where both absorption (orange) and dispersion (blue) are plotted. For maximum incident microwave power of 3 W, saturation effects result in the spectrum shown in Supplementary Fig. 9b. As clearly visible, the experimental line shapes did not reproduce the single homogenous line expected for BDPA. We attribute the inhomogeneous broadening to the number of grains in this particular experimental series at microwave powers up to 3 W.

Information of the microwave field strength B_1 becomes possible upon quantitative analysis of EPR spectra, which includes the relaxation times T_1 and T_2. While homogeneous lines can be analyzed based on analytical prescriptions,[8] the approach becomes more complicated in presence of the inhomogeneous broadening at hand.[9] Accordingly, the inhomogeneous broadening underlying experimental spectra has been deconvoluted numerically.

The deconvolution was calculated with the dispersion spectrum of the low-power spectrum in Supplementary Fig. 9a. Technically, the inhomogeneous distribution function of homogeneous lines with $T_1 = T_2 = 70$ ns was computed by a Tikhonov regularization using the python package lsmr provided in scipy.sparse.linalg. The regularization parameter of 0.5 was selected from an L-curve approach.[10] The resultant distribution function is shown in olive in Supplementary Fig. 9c. For spectrum simulations, only the non-negative values were considered (purple curve underneath olive), whereas negative values were set to zero.

The spectra simulated in this way are superimposed as dashed lines in Supplementary Figs. 9a and b. The low-power dispersion spectrum virtually coincides with the simulations (dashed red), which is not surprising since this experimental spectrum was used to calculate the underlying distribution function. For the complementary experimental low-power absorption spectrum, the simulation (dashed green) is in good overall agreement. For the high-power spectra in Supplementary Fig. 9b, best overall agreement between simulated and experimental data was found for B_1 = 81 µT.

A complementary estimate for B_1 was extracted from the power-progressive peak-to-peak amplitude y_{pp} of the CW absorption spectrum. Supplementary Fig. 9d shows experimental data points (orange) for a variety of driving amplitudes as well as a simulation based on the distribution function (green) in Supplementary Fig. 9c. Best agreement between the experimental and simulated curves when tying the data point at the highest power of 3 W to B_1 = 120 µW and allowing for an overall scaling factor of 0.956 to experimental data.

In summary, the analysis of power-progressive CW spectra suggest B_1 amplitudes on the order of 100 µT for the largest incident power of 3 W. Taking the lower estimate of 81 µT, we thus conclude that the 7.5 GHz resonator achieves a power-to-field conversion factor Λ[11] on the order of 50 $\mu T/\sqrt{W}$. Note

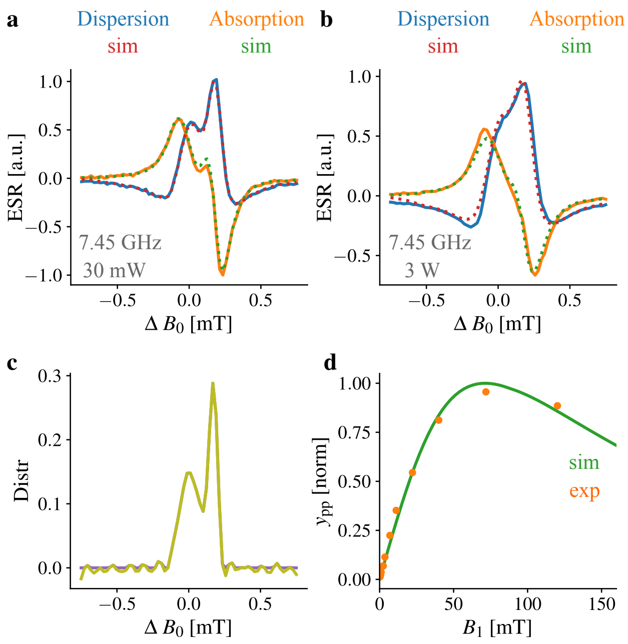

Supplementary Figure 9. Power saturation on BDPA at 7.45 GHz to estimate the microwave field strength. **(a)** CW EPR derivative spectrum with 30 mW of microwave drive, showing experimental dispersion (blue) and absorption (orange) and simulations (dashed) with deliberately weak B_1 (1 μT) for the line shape in the linear regime. **(b)** CW EPR derivative spectrum with 3 W microwave drive, with coloring as in panel (a). The simulation was performed with $B_1 = 81$ μT for best possible agreement with the experimental spectrum. **(c)** Inhomogeneous distribution used to model inhomogeneous broadening, where the homogeneous line had $T_1 = T_2 = 70$ ns. The olive distribution function was retrieved numerically by deconvolution of the experimental spectrum in panel (a) with a regularization parameter of 0.5, while the non-negative purple curve underneath was used for spectral simulation. **(d)** Progressive power saturation showing normalized peak-to-peak amplitude y_{pp} of the EPR absorption spectrum as a function of the driving strength B_1 for simulated (blue) and experimental (orange) data. A reasonable overlap between experimental and simulated data was achieved for $B_1 = 120$ μT at 3 W and an overall scaling factor of 0.956 to y_{pp}.

that these field amplitudes, including Λ, denote linearly polarized excitation fields, as encompassed in the underlying equations for CW EPR.[8]

While these experiments were performed on a separate setup at ambient temperature and pressure, we have further experimental evidence from pulsed EPR inside the X-Treme endstation. While the 7.5 GHz resonators are outside the frequency range of the pulsed EPR setup described in Supplementary Note 7, the resonance frequency can be moved from 7.5 GHz to 4.25 GHz by exchanging the three spacers fabricated from teflon (see Supplementary Fig. 1a) by spacers fabricated from sapphire. Since the down-shift in frequency is mainly related to the concentration of electric fields inside the sapphire spacers, one would expect that such a down-tuned resonator has a comparable conversion factor Λ.

Pulsed EPR of BDPA inside the X-Treme endstation at the lowest possible sample temperature revealed that a 100 ns long pulse achieved a π rotation for an incident power on the order of 25 W, which corresponds to a Rabi oscillation frequency of 5 MHz. The linearly polarized magnetic field related to this Rabi frequency has an amplitude of 355 μT. With these parameters, a power-to-field conversion factor of $\Lambda = 71$ μT/$\sqrt{\text{W}}$ is obtained, which is on the same order of magnitude as the estimation of 50 μT/$\sqrt{\text{W}}$ from CW EPR.

(intentional page break)

SUPPLEMENTARY NOTE 9. THEORY ON G-FREE PEAK

This section provides the theoretical foundation for our interpretation of the g_{free} peak, namely its relation to a coupled spin that is not directly probed with element-selective X-ray detection. We consider a pair of electron spins S_1 and S_2, which are either coupled via exchange coupling or dipolar coupling. The longitudinal magnetic moment $\hat{\mu}_{1,z}$ of spin S_1 is the observable that is probed by element-specific XDEPR. The magnetic moment $\hat{\mu}_{1,z}$ is directly proportional to the longitudinal spin projection $\hat{S}_{1,z}$ of spin S_1. Since we are mainly interested in relative changes of $\hat{\mu}_{1,z}$ caused by resonant microwave irradiation, we base our analysis on the expectation value of the spin projection $\langle \hat{S}_{1,z} \rangle$. In this way, we can omit prefactors related to the thermal polarization of the spin system. The equilibrium spin state has therefore $\langle \hat{S}_{1,z} \rangle = 1$ and resonant irradiation will result in $\langle \hat{S}_{1,z} \rangle < 1$.

When observing solely $\langle \hat{S}_{1,z} \rangle$, one might expect that one is *blind* to S_2. However, as we will outline below, this is not the case if the spins S_1 and S_2 become indistinguishable, e.g. when (non-secular) coupling terms dominate the spin Hamiltonian and mix the spin states.

For exchange-coupled spins with isotropic coupling strength J, the rotating-frame spin Hamiltonian in units of angular frequency can be written as[12]

$$\begin{aligned}\mathscr{H}_J = -\delta/2 \cdot \hat{S}_{1,z} + \delta/2 \cdot \hat{S}_{2,z} + J \cdot \hat{S}_{1,z} \cdot \hat{S}_{2,z} \\ + J \cdot (\hat{S}_{1,x} \cdot \hat{S}_{2,x} + \hat{S}_{1,y} \cdot \hat{S}_{2,y})\end{aligned} \tag{1}$$

where $\hat{S}_{i,j}$ is the spin operator of spin i projected along Cartesian coordinates $j = (x,y,z)$. The variable δ is the frequency separation between the spins, such that the reference frame rotates at the frequency that is centered between the Zeeman frequencies of S_1 and S_2. Such a spin pair is said to be in the strong coupling limit if $J > \delta$. In this case, the off-diagonal elements in $\mathscr{H}_J$, e.g. the second line in Eq (1), cause the spins S_1 and S_2 to become non-separable (indistinguishable), since the eigenstates are no longer linear combinations of single-spin states. In the weak coupling limit for $J \ll \delta$, on the contrary, the eigenstates are linear combinations of single-spin states and the spins thus distinguishable from each other.

For a spin pair with dipolar coupling of strength A, we consider the simplified Hamiltonian that is commonly applied in EPR spectroscopy[13],

$$\begin{aligned}\mathscr{H}_A = -\delta/2 \cdot \hat{S}_{1,z} + \delta/2 \cdot \hat{S}_{2,z} + A \cdot \hat{S}_{1,z} \cdot \hat{S}_{2,z} \\ - A/2 \cdot (\hat{S}_{1,x} \cdot \hat{S}_{2,x} + \hat{S}_{1,y} \cdot \hat{S}_{2,y})\end{aligned} \tag{2}$$

Also in this case, strong coupling is attained if $A > \delta$, where the off-diagonal terms in the second line of Eq (2) become relevant.

While the exchange coupling J is proportional to the overlap between the wavefunctions of the electrons that carry the spin S_1 and S_2, the dipolar coupling A is obtained from

$$A = 2\pi \cdot c_{\text{dd}}/r^3 \cdot (1 - 3 \cdot \cos^2\theta) \tag{3}$$

$$c_{\text{dd}} = \frac{\mu_0}{4\pi} \cdot \gamma_e^2 h \tag{4}$$

where r is the inter-spin distance, θ the angle between the inter-spin vector and the external magnetic field along the z axis, γ_e the gyromagnetic ratio in frequency units, and h the Planck constant. The constant c_{dd} evaluates to approximately 52 MHz·nm^3 and determines the order of magnitude of A for a given distance r.

In order to compute spectra numerically, we added the time-dependent driving term

$$\mathscr{H}_{\text{drive}}(t) = \omega_1 \cdot \cos(\Omega t) \cdot (\hat{S}_{1,x} + \hat{S}_{2,x}) \tag{5}$$

where Ω is the frequency detuning between the drive and the spins, and $\omega_1 = \pi\gamma_e B_1$ is the strength of the drive. Simulations were performed with the SPin DYnamics ANalysis (SPIDYAN) package[14] and we have used the same relaxation and drive parameters as for the spectra in the main text in Fig. 2b. Accordingly, we included relaxation times T_1 = 1 µs and T_2 = 200 ns for all the transitions of the considered two-spin system and we fixed the amplitude of the driving field to B_1 = 100 µT. The steady state for this spin system was obtained by time-evolution for 10 µs, where relaxation transients decayed. The steady-state polarization of each spin was obtained by computing the expectation value $\langle S_{1,z} \rangle$ and $\langle S_{2,z} \rangle$. Setting either $J = 0$ in $\mathscr{H}_J$ or $A = 0$ in $\mathscr{H}_A$ reproduced the analytical single-spin steady-state solution of the Bloch equations.

Simulated spectra for exchange coupling and dipolar coupling are shown in Supplementary Fig. 10a and b, respectively. The spin-selective polarization of S_1 is shown in blue, whereas the orange curve shows the spectrum when detecting both spins together, adjusted to a comparable y-axis.

The spectra of both spins show the expected doublets centered around the resonance frequencies $\Omega = \pm\delta/2$. These spectra are symmetric and the evolution of the peak positions as a function of the frequency separation δ/J and δ/A is given in magnetic resonance literature[12]. For spin-selective detection, the spectra exhibit principally the doublet of S_1, but there is also a relevant contribution from the doublet of S_2. Accordingly, a signature of S_2 is observed in the spin-selective spectrum of S_1, meaning that spin-selectivity does not inhibit detection of coupled spins. The underlying reason is that the frequency separation δ between the spins is comparable to the coupling strength, such that S_1 and S_2 cannot be distinguished.

We quantify the resultant leakage of the coupled spin S_2 to the spectrum of S_1 with the variable l_{spec}, as indicated in green in Supplementary Fig. 10a and b. The dependence of l_{spec} as a function of the frequency separation δ between the spins is shown in Supplementary Fig. 10c and d for exchange and dipolar coupling, respectively. Simulations for three different numerical coupling values are shown and colored according to the legend. For the largest separations δ, all the curves decay to zero since leakage becomes negligible in the weak coupling regime, where the spins are fully distinguishable. For the smallest separations δ in the strong coupling regime, leakage is more pronounced for exchange coupling than for dipolar coupling. This is due to the reduction of the spectrum with exchange coupling into one single peak for $\delta = 0$, whereas two peaks result with dipolar coupling. Moreover, also for larger δ, leakage generally appears more pronounced for exchange coupling as compared to dipolar coupling. This is in-

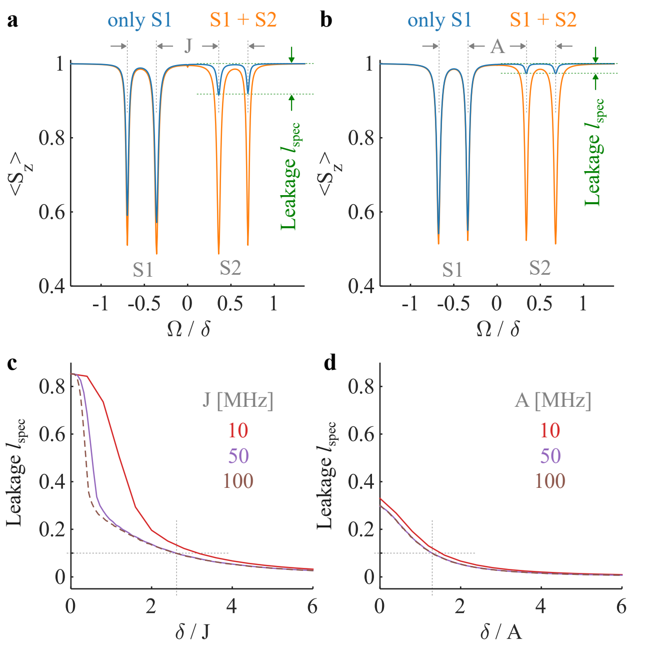

Supplementary Figure 10. Spin-selective polarization of two coupled spins S_1 and S_2. **(a,b)** Spectra of spin-selective polarization $\langle S_{1,z}\rangle$ (blue) and non-selective polarization $\langle S_{1,z}\rangle + \langle S_{2,z}\rangle - 1$ (orange) with exchange coupling J in panel (a) and dipolar coupling A in panel (b). The leakage of S_2 into $\langle S_{1,z}\rangle$ is indicated by l_{spec} (green). The numerical values for the simulation were $A = J = \delta/3 = 2\pi\cdot$ 50 MHz, T_2 = 200 ns, T_1 = 1 µs, and B_1 = 100 µT. **(c,d)** Dependence of leakage l_{spec} on frequency separation δ for exchange coupling J in panel (c) and for dipolar coupling A in panel (d) with coupling strengths according to color coding in the legend.

dicated by the dashed gray lines, which intersect the y-axis at l_{spec} = 0.1, but at fairly different normalized separations δ on the x-axis.

When comparing the different coupling strenghts, the smallest coupling of 10 MHz (red) displays significantly larger leakage than the 50 MHz (solid violet) and 100 MHz (dashed brown) data. This is attributed to spectral broadening by the drive ω_1, which becomes comparable to δ, J and A for the smallest coupling of 10 MHz. For the larger couplings, on the contrary, ω_1 is much smaller compared to the coupling strength and the 50 MHz and 100 MHz data virtually overlap over a large range.

To outline how leakage can give rise to a g_{free} peak in the case of CuPc spins coupled to trapped electrons, the above spin-selective polarization patterns were convoluted with the corresponding spectral weighting factors. The simulated spectra are displayed in Supplementary Fig. 11a, which shows CuPc (orange) and the g_{free} peak (green) related to the coupled electron. The spectral amplitudes are chosen such that CuPc and trapped spins are in a 1:1 ratio. The CuPc spectrum has already been shown in the main text and accounts for dipolar interactions between CuPc centres by phenomenological Gaussian broadening. The employed broadening by 2.5 mT corresponds to roughly 75 MHz in frequency units. Under the assumption that the coupled electron spin is at rest,

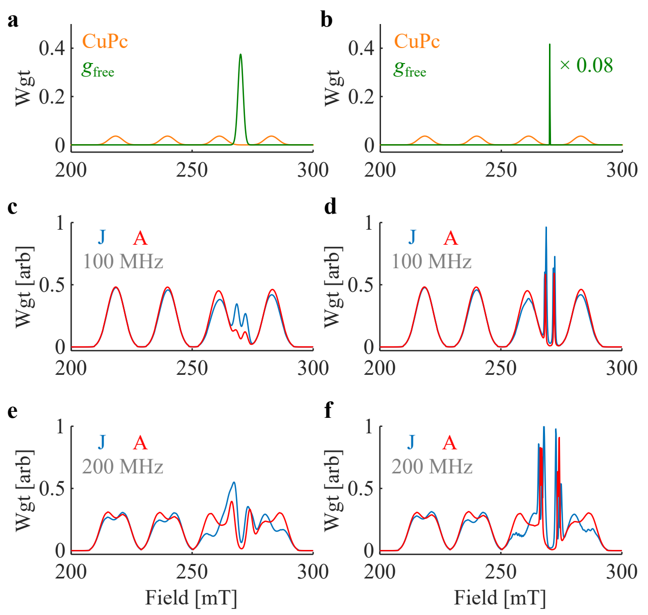

Supplementary Figure 11. Representative simulations for CuPc spins coupled to trapped electrons. **(a,b)** Spectral distribution of CuPc (orange) and trapped electron at g_{free} (green) with either 2.5 mT broadening in panel (a) or 0.1 mT in panel (b). **(c,e)** Resulting spin-selective polarization of CuPc for the spectra in panel (a) for either exchange coupling J (blue) or dipolar coupling A (red) with coupling strength of 100 MHz in panel (c) and 200 MHz in panel (e). **(d,f)** Corresponding simulations for spectra in panel (b) with coupling strength of 100 MHz in panel (d) and 200 MHz in panel (f). The field axis in all panels has a step size of 143 µT. The equivalent frequency-domain grid has 4 MHz step size and was used to calculate panels (c-f) by summing pre-calculated hole patterns $1 - \langle S_{1,z}\rangle$ at appropriate δ, Ω, and spectral weighting factors.

the same line broadening has been applied to the g_{free} peak.

Resultant element-specific CuPc spectra for coupling strengths of 100 MHz and 200 MHz are shown in Supplementary Figs. 11c and e, respectively. The computations were performed for exchange-coupled spins (blue) and for dipole-coupled spins (red). All spectra show an extra contribution at g_{free} related to the coupled spin. The g_{free} doublet peak is more pronounced for exchange coupling than for dipolar coupling, in accordance to the larger leakage l_{spec} observed above in Supplementary Fig. 10. For the 200 MHz coupling, the splitting of the CuPc hyperfine lines into a doublet can also be inferred, since the coupling strength exceeds the linewidth.

We found the amplitude of the resultant g_{free} doublet to depend strongly on the spectral linewidth of the g_{free} peak as well as on the spectral overlap between the CuPc and the g_{free} spectrum. A representative narrow-line scenario is shown in Supplementary Fig. 11b, where the g_{free} peak (green) has a narrow linewidth of 0.1 mT. Element-specific CuPc spectra for this case are shown in Supplementary Figs. 11d and f for couplings of 100 MHz and 200 MHz, respectively. The resultant doublets around g_{free} are much more pronounced in this narrow-line case.

Indeed, there is the possibility that the linewidth of the

$g_{\rm free}$ peak narrows below the linewith of the CuPc spectrum, namely via motional narrowing due to delocalization of the electron over several Pc molecules. Evidence for such delocalization has been provided for light-induced EPR signals in H_2Pc at $g_{\rm free}$, where narrowed lines have been observed[15,16]. The motion of the coupled electron due to delocalization as well as its longitudinal relaxation time T_1 would then also influence the effective spin-spin coupling J or A between the Pc radical and the metal centre due to averaging of that interaction. For dynamic components with timescales that are faster than the inverse coupling, the spin-spin couplings are averaged to zero, which gives rise to narrowed spectral lines, hence the notion of motional narrowing[12]. For the simulations displayed above in Supplementary Fig. 10a and b with ordinary non-selective detection (orange), motional narrowing would reduce the doublets associated to S_1 and S_2 to a single line each. The two coupled spins appear thus decoupled based on the eigenvalues of the spin Hamiltonian that determine the spectrum. The extent of spectral leakage $l_{\rm spec}$ in spin-sepective spectra does, however, depend on separability of the eigenstates of the spins. It is important to point out that it is the time-averaged coupling that goes to zero, while the spins remain coupled at all times. We would thus expect a non-zero leakage $l_{\rm spec}$ in the limit of motional narrowing and thus a single-peak contribution at $g_{\rm free}$, as observed experimentally. The actual influence of motional narrowing on $l_{\rm spec}$ would require numerical simulations that are beyond the scope of the current manuscript.

Other than motional narrowing, we would also expect a single-peak contribution at $g_{\rm free}$ in spin-selective spectra for slower dynamics. The relevant regime is known as motional broadening and attained for dynamics that are below the inverse coupling strength[12]. In this regime, the coupling is not yet fully averaged to zero and a broadened single-peak contribution appears instead of the doublets that are obtained with the static simulations shown above in Supplementary Figs. 10 and 11.

Since the narrow-line scenario in Supplementary Fig. 11 results in a dominant $g_{\rm free}$ contribution as observed experimentally, it is worth to mention another possibility than motional averaging for a narrow linewdith of the $g_{\rm free}$ peak. Essentially, a narrow-line $g_{\rm free}$ peak would be the point of departure for a more rigorous treatment of line broadening than the phenomenological Gaussian broadening applied here. The line-broadening is caused by spin-spin interactions and any interaction between CuPc and trapped electrons will result in a finite leakage $l_{\rm spec}$ that is omitted in the phenomenological model. However, in analogy to motional narrowing mentioned above, more elaborate modeling is far beyond the scope of the present manuscript, as it would involve more than two coupled spins. The analysis presented here for a pair of spins serves mainly as a model to corroborate the interpretation of the $g_{\rm free}$ peak in element-specific spectra.

Despite these simplifications, it is instructive to relate the spin-spin couplings of 100 - 200 MHz used in Supplementary Fig. 11 to the experimentally investigated samples. Dipolar couplings of 100 - 200 MHz correspond to spins with distances in the lower nanometre regime. For oriented Pc samples[17], the distances between adjacent Pc molecules are on the order of 1.26 nm along the molecular plane, such that A can reach 25 MHz between adjacent Pcs within the same plane. The distances between Pcs on adjacent layers is 0.37 nm, such that A can reach 1 GHz for the closest neighbors. At such close distances, one would also expect a contribution from exchange coupling due to wavefunction overlap, as for instance from the ligands π orbitals of next-layer Pcs. To the best of our knowledge, there are no literature values for exchange couplings between metal-loaded Pcs to radicals on an adjacent Pc ring. However, experimental data are available for the coupling of VOPc and CuPc spins to an electron trapped on its ring and estimated J couplings are all much larger than 10 GHz[18,19]. As discussed in the main text, we ascribe such localized trapping sites to the loss of the XDEPR signal at higher photon flux, whereas the $g_{\rm free}$ peak is attributed to delocalized trapping sites, which will have smaller J due to the larger spatial extent of the wavefunction.

The concentration of trapped electrons can be estimated based on

$$c_e = (f_{\rm ph}/t_{\rm film} \cdot \alpha) \cdot N_e \cdot \tau_e \tag{6}$$

where the quantity in brackets is the rate of absorbed X-ray photons per volume, calculated from the incoming photon flux $f_{\rm ph}$, the sample thickness $t_{\rm film}$ and the fraction of absorbed photons α, which evaluates to roughly 3 ph/ms/μm^3. On average, each photon creates N_e secondary electrons with lifetime τ_e. With $N_e = 500$, a representative number for insulators[20], the mean lifetime of trapped, secondary electrons, needs to be about one minute in order to attain a 1:1 ratio between the trapped electrons and CuPc spins that are diluted by 5% into Pc. Note that such a 1:1 ratio has been assumed in the simulations above. A 1:1 ratio is also adequate when considering the experimental results with a ten times larger photon flux in Supplementary Note 4, where the concentration c_e would become comparable to the concentration of Pc molecules. We consider the resultant excess of secondary electrons to be responsible for the population of localized trapping sites that result in the loss of the XDEPR signal at the larger photon flux.

With respect to the lifetime τ_e, we expect fairly different timescales to contribute to the effective lifetime τ_e due to the heterogeneity of trapping sites. Lifetimes of trapped electrons can be inferred from studies of photo-excited EPR in H_2Pc. It has been noted that CuPc as an impurity inside H_2Pc does not alter the resultant trap density at room temperature[15]. At a temperature of 100 K, lifetimes on the order of minutes can be inferred from the reported cessation kinetics upon light irradiation[16]. Moreover, short-lived photo-excited radicals with lifetimes on the order of 10 μs were observed for temperatures around 100 K[21,22]. Since we would expect these lifetimes to be prolonged at the temperatures in our study, trap concentrations that are comparable to the metal centre concentration do indeed appear conceivable. As discussed in the main text, we also consider such a network of trapped electrons to be efficient for mediation of longitudinal spin relaxation. Especially short-lived secondary electrons cause fluctuations that can be transmitted via the coulomb interaction

among trapped radicals.

In summary, we have detailed in this Section the theory that underlines our interpretation of the g_{free} peak that arises form the non-separability of coupled spin states. From our analysis, we conclude that the dominant g_{free} peak observed experimentally can be explained by trapped secondary electrons at a concentration that is comparable to the number of metal centres. Due to the extended π orbitals of the Pc rings, the trapped electrons are not at rest, but constitute a dynamic metastable network of spins that are coupled to the metal centres. These dynamics result in motional averaging of the spins-spin couplings, which we consider essential for observation of a single peak centered at g_{free}. Furthermore, dynamic components on picosecond timescales will promote longitudinal spin relaxation. From our analysis, it is also follows that the signal at g_{free} might be more intense than the signal of the metal center, as experimentally observed, motivating our choice to also monitor the g_{free} peak dependence on the external parameters. However, this choice does not hide the proof of concept that XEDPR is indeed possible, regardless of the presence or not of the free radicals. Future efforts on XDEPR should focus on experimental schemes that minimize the concentration of trapped secondary electrons.

REFERENCES FOR SUPPLEMENTARY INFORMATION

[1] A. Doll, "Pulsed and continuous-wave magnetic resonance spectroscopy using a low-cost software-defined radio," AIP Adv. **9**, 115110 (2019).

[2] F. James, M. Winkler, *et al.*, "Minuit user's guide," CERN, Geneva **23** (2004).

[3] J. Pescia, "La mesure des temps de relaxation spin-réseau très courts," Ann. phys. **13**, 389–406 (1965).

[4] V. A. Atsarkin, V. V. Demidov, G. A. Vasneva, B. M. Odintsov, R. L. Belford, B. Radüchel, and R. B. Clarkson, "Direct Measurement of Fast Electron Spin-Lattice Relaxation: Method and Application to Nitroxide Radical Solutions and Gd^{3+} Contrast Agents," J. Phys. Chem. A **105**, 9323–9327 (2001), Note on integration of analytical solutions: Since the time-dependence is a simple oscillation at ω_{mod}, integration was accomplished by division of the reported analytical formulas by ω_{mod}, namely by division of the real and imaginary parts of the oscillation amplitudes in equations (9) and (10) by ω_{mod}.

[5] J. Granwehr, J. Leggett, and W. Köckenberger, "A low-cost implementation of EPR detection in a dissolution DNP setup," J. Magn. Reson. **187**, 266–276 (2007).

[6] G. Bodenhausen, R. Freeman, and D. L. Turner, "Suppression of artifacts in two-dimensional J spectroscopy," J. Magn. Reson. **27**, 511 – 514 (1977).

[7] M. Warner, S. Din, I. S. Tupitsyn, G. W. Morley, A. M. Stoneham, J. A. Gardener, Z. Wu, A. J. Fisher, S. Heutz, C. W. M. Kay, and G. Aeppli, "Potential for spin-based information processing in a thin-film molecular semiconductor," Nature **503**, 504–508 (2013).

[8] R. D. Nielsen and B. H. Robinson, "The effect of field modulation on a simple resonance line shape," Concepts magn. reson. **23A**, 38–48 (2004).

[9] A. Lund, E. Sagstuen, A. Sanderud, and J. Maruani, "Relaxation-Time Determination from Continuous-Microwave Saturation of EPR Spectra," Radiat. Res. **172**, 753–760 (2009).

[10] P. Johnston and R. Gulrajani, "Selecting the corner in the L-curve approach to Tikhonov regularization," IEEE. Trans. Biomed. Eng. **47**, 1293–1296 (2000).

[11] J. S. Hyde, W. Froncisz, and T. Oles, "Multipurpose loop-gap resonator," J. Magn. Reson. **82**, 223–230 (1989).

[12] M. H. Levitt, *Spin dynamics: Basics of nuclear magnetic resonance* (John Wiley & Sons, 2001).

[13] P. P. Borbat and J. H. Freed, "Pulse Dipolar Electron Spin Resonance: Distance Measurements," in *Structural Information from Spin-Labels and Intrinsic Paramagnetic Centres in the Biosciences* (Springer, 2013) pp. 1–82.

[14] S. Pribitzer, A. Doll, and G. Jeschke, "SPIDYAN, a MATLAB library for simulating pulse EPR experiments with arbitrary waveform excitation," J. Magn. Reson. **263**, 45–54 (2016).

[15] J. R. Harbour and R. O. Loutfy, "An electron spin resonance investigation into dark and light-induced paramagnetism in metal-free phthalocyanines," J. Phys. Chem. Solids **43**, 513–520 (1982).

[16] R. L. Sasseville, J. R. Bolton, and J. R. Harbour, "Detection of a new photoinduced electron paramagnetic resonance signal in particle dispersions of metal-free .alpha.-, .beta.- and x-phthalocyanine," J. Phys. Chem. **87**, 862–867 (1983).

[17] A. Hoshino and H. Miyaji, "Redetermination of the crystal structure of a-copper phthalocyanine grown on KCl," . B (2003).

[18] D. V. Konarev, A. V. Kuzmin, S. S. Khasanov, M. S. Batov, A. Otsuka, H. Yamochi, H. Kitagawa, and R. N. Lyubovskaya, "Salts with titanyl and vanadyl phthalocyanine radical anions. Molecular design and effect of cations on the structure and magnetic and optical properties," CrystEngComm **20**, 385–401 (2018).

[19] M. A. Faraonov, I. A. Yakushev, E. I. Yudanova, M. Pelmus, S. M. Gorun, A. Otsuka, H. Yamochi, H. Kitagawa, and D. V. Konarev, "Synthesis, X-ray Structures, and Optical and Magnetic Properties of Cu(II) Octafluoro-octakisperfluoro(isopropyl)phthalocyanine: The Effects of Electron Addition and Fluorine Accretion," Inorg. Chem. **62**, 11390–11401 (2023).

[20] J. Cazaux, "Correlation between the x-ray induced and the electron-induced electron emission yields of insulators," J. Appl. Phys. **89**, 8265–8272 (2001).

[21] R. L. Sasseville, A. R. McIntosh, J. R. Bolton, and J. R. Harbour, "A flash photolysis-electron paramagnetic resonance study of light-generated paramagnetic charge carriers in metal-free .alpha.-, .beta.- and x-phthalocyanines," J. Phys. Chem. **87**, 868–872 (1983).

[22] R. L. Sasseville, A. R. McIntosh, J. R. Bolton, and J. R. Harbour, "Flash photolysis electron paramagnetic resonance studies of charge-carrier production in sublimed films of phthalocyanine," J. Phys. Chem. **88**, 3139–3142 (1984).
